# Validation of the teaching interpersonal style questionnaire in physical education

**DOI:** 10.3389/fpsyg.2025.1702118

**Published:** 2025-11-21

**Authors:** Francisco M. Leo, David Sánchez-Oliva, Javier Fernandez-Rio, Miguel A. López-Gajardo, Juan J. Pulido

**Affiliations:** 1 University of Extremadura, Cáceres, Spain; 2 Universidad de Oviedo, Oviedo, Spain; 3 Universidad de Extremadura, Badajoz, Spain

**Keywords:** basic psychological needs, need support, need thwarting, psychometric properties, self-determination theory

## Abstract

**Introduction:**

Although research in Physical Education (PE) has widely examined the role of teachers’ motivational style, there is still a lack of validated instruments that jointly assess both the supportive and thwarting dimensions of interpersonal behaviors. This gap limits progress in understanding how teachers can either foster or undermine students’ motivational processes in PE. Thus, this study aimed to provide evidence of validity for the Teachers’ Interpersonal Style Questionnaire in Physical Education (TISQ-PE) as a multidimensional measure of students’ perceptions of their teachers’ interpersonal behaviors.

**Method:**

A total of 54 Primary and Secondary education students participated (*M*_age_ = 11.96 ± 1.95; 337 girls).

**Results:**

A confirmatory factor analysis provided evidence of factorial validity, supporting the six correlated main factors of the scale (autonomy-support, competencesupport, relatedness-support, autonomy-thwarting, competence-thwarting, and relatedness-thwarting). Evidence of convergent validity was supported by satisfactory average variance extracted values for all factors. Furthermore, the TISQ-PE provided evidence of discriminant and nomological validity and showed invariance by sex and educational stage. Lastly, all factors showed acceptable internal consistency values.

**Conclusion:**

Therefore, the TISQ-PE offers evidence of validity and reliability to assess students’ perceptions of their teachers’ interpersonal style in PE class, offering researchers and practitioners a comprehensive instrument to better understand both supportive and thwarting teaching behaviors.

## Introduction

Current pedagogical approaches require students’ active involvement in the teaching-learning process (Behzadnia et al., 2018; Ommundsen and Kvalø, 2007). Student-teacher and student-student interactions create interpersonal and multidirectional relationships that can impact students’ learning positively or negatively (Behzadnia et al., 2018). Focusing on the student-teacher interaction, students’ perception of the teacher’s interpersonal style, including supporting and thwarting interpersonal behavior (Van den Berghe et al., 2013), could help to understand class environments and their outcomes.

From the perspective of self-determination theory (SDT; Deci and Ryan, 2000), teachers’ behaviors play a key role in shaping students’ motivational processes and educational outcomes. Teachers can implement a range of interpersonal behaviors, some of which support students’ autonomy, competence, and relatedness, while others may thwart these basic psychological needs. These supportive and thwarting teaching behaviors are distinct sets of practices that can coexist within the same classroom (Haerens et al., 2015; Van den Berghe et al., 2016). Specifically, autonomy-supportive teaching styles are characterized by the use of strategies that encourage democratic leadership. In this atmosphere, students can feel themselves to be the protagonists of the activity they perform, not only while performing the activity, but also during the decision-making and supervision processes (Reeve, 2009). Competence-supportive teaching styles are characterized by: (a) offering students challenging activities that match their ability level, (b) expressing confidence in their capacity to effectively engage in the activity; (c) showing effective models before task participation, (d) providing encouragement and specific help during activity engagement, (e) offering positive feedback and sincere praise after successful task completion, and (f) avoiding critical and demeaning feedback after poor performances or mistakes (Jang et al., 2010). Finally, relatedness-supportive teaching strategies are defined by the level of empathy shown in the teacher-student relationship (Haerens et al., 2015). Teachers should try to help students feel socially connected and fully internalize the value/consequence of their behaviors (Van den Berghe et al., 2013).

On the other hand, an autonomy-thwarting teaching behavior is characterized by: (a) frequent use of directive and intimidating behaviors, (b) the adoption of a position of authority when desired attributes or behaviors are not displayed by the subordinates, (c) excessive personal control during the supervision of the tasks, and (d) coercive and pressure behaviors with imperative input of instructions to execute a skill or ability (Assor et al., 2004; Reeve, 2009). A competence-thwarting teaching behavior is characterized by: (a) public critical feedback, normative and externally referenced comparison, (b) activities that prevent students from setting individualized and attainable goals that stimulate personal self-improvement and foster progress, (c) a chaotic class climate where objectives, expectations, and rules are unclear (Van den Berghe et al., 2016). Finally, teachers thwart students’ need for relatedness by being unfriendly or even rejecting and excluding students, producing an emotionally cold environment (De Meyer et al., 2014).

These supportive and thwarting teaching behaviors can shape students’ motivational processes by influencing the satisfaction or frustration of their basic psychological needs (Deci and Ryan, 2000). Within SDT, three needs are essential to understanding the *what* (i.e., content) and *why* (i.e., process) of goal pursuits: “*innate psychological nutriments that are essential for ongoing psychological growth, integrity, and wellbeing*” (Deci and Ryan, 2000, p. 229). These three basic psychological needs are: autonomy (i.e., the feeling of being able to make decisions without pressure and guiding one’s own behavior), competence (i.e., the feeling of efficiency in the task or the ability to perform), and relatedness (i.e., the feeling of belongingness and connectedness to other individuals, groups or cultures) (Deci and Ryan, 2000). These basic psychological needs can be either satisfied or frustrated depending on the teacher’s interpersonal behaviors (Deci and Ryan, 2000). Supportive behaviors provide students with opportunities for choice, guidance, and meaningful social connection, fostering the satisfaction of their needs (Ahmadi et al., 2023). Conversely, thwarting behaviors undermine students’ sense of autonomy, competence, and relatedness, leading to need frustration (Ahmadi et al., 2023). In turn, the fulfillment or frustration of these needs directly influences students’ motivation and outcomes in the PE context (Deci and Ryan, 2000).

Specifically, SDT research has consistently highlighted the importance of teachers’ interpersonal style for students’ satisfaction and/or frustration of their basic psychological needs and for their motivation (Howard et al., 2025; Vasconcellos et al., 2020). Students’ perceptions of a need-supportive teaching style have been positively related to need satisfaction and self-determined motivation, which in turn are associated with higher enjoyment, greater engagement, persistence in learning activities, wellbeing, and reduced feelings of boredom, ill-being, or dropout (Haerens et al., 2015; Howard et al., 2025; Vasconcellos et al., 2020; Wang et al., 2024). Conversely, perceptions of a need-thwarting teaching style have been associated with need frustration, lower self-determined motivation, and negative outcomes such as disengagement, boredom, ill-being, and avoidance behaviors (Assor et al., 2005; Haerens et al., 2015; Howard et al., 2025; Vasconcellos et al., 2020). These findings illustrate how teachers’ motivational styles can directly and indirectly shape students’ emotional, cognitive, and behavioral responses within the PE context.

Based on the SDT, several instruments have been designed to assess the interpersonal style adopted by teachers during the Physical Education (PE) lessons. The Questionnaire of Basic Psychological Needs Support in PE (QBPNS-PE; Sánchez-Oliva et al., 2013) is a 12-item instrument to measure autonomy-, competence-, and relatedness-support. Another interesting instrument is the Motivational Climate in PE Scale (MCPES; Soini et al., 2014). This scale is composed of four factors (18 items) supporting perceived autonomy, social relatedness, task involvement, and ego involvement. This instrument was adapted to Spanish from the teachers’ point of view (Need-Supportive Teaching Style Scale; Abós et al., 2018) and shows adequate factorial structure and internal consistency. Similarly, the Need-Supportive Teaching Style Scale in PE (NSTSSPE; Liu and Chung, 2017) is another instrument designed to assess students’ perceptions of a need-supportive teaching style. The results yielded a three-factor structure of the NSTSSPE: structure (i.e., competence support), involvement (i.e., relatedness support), and autonomy support. Evidence of nomological validity was also supported, and weak, strong, and strict measurement invariance was evidenced across gender, grade, and time, respectively. All these instruments have in common the assessment of the “bright” or positive side of motivation (Ryan and Deci, 2000): task climate support, ego climate support, autonomy support, and relatedness support. Less attention has been paid to the “dark” side of teaching practices although it is acknowledged that the presence of need-thwarting teaching behaviors is more than the mere absence of need support (Bartholomew et al., 2011; De Meyer et al., 2014). Therefore, there is a need to develop assessment instruments that include a holistic measurement of teaching styles, both supportive and thwarting (Van den Berghe et al., 2016).

In line with this idea, the adapted version for PE teachers of the Empowering and Disempowering Motivational Climate Questionnaire (EDMCQ-PE: Milton et al., 2018) was developed. It assesses students’ perceptions of the class climate and includes 31 items grouped into five factors: task-involving, autonomy-support, relatedness-support, ego-involving, and controlling. Regrettably, it does not include a relatedness-thwarting dimension. Furthermore, the task- and ego-involving factors included, rooted in motivational climate theories (i.e., Achievement Goal Theory; Ames, 1992; Nicholls, 1984), may be related to the positive and negative dimensions of competence (i.e., in SDT). Therefore, a scale considering exclusively the tridimensional need-supportive and need-thwarting factors, grounded in the SDT, seems to be needed (Appleton et al., 2016).

Therefore, despite the quality of the above-mentioned instruments, all of them present some limitations that justify the development of a new and specific questionnaire to assess students’ perceptions of their teachers’ interpersonal style in the primary and secondary education contexts. To our knowledge, there is no scale exclusively based on the SDT that assesses full spectrum of teachers’ interpersonal behaviors (including the “bright” and “dark” sides of motivation). In addition, as noted by Appleton et al. (2016), it would be interesting to create balanced instruments (brief and integral), as no psychometric attempts have been made to produce a relatively short and systematic scale that includes students’ perceptions of the teachers’ supporting and thwarting interpersonal style. Also, it is necessary to assess the invariance across gender and education level to ensure that the instrument can be used with each subgroup (i.e., boys and girls; primary and secondary education). Although some scales that assessed students’ perception of the interpersonal teaching style did not examine measurement invariance (i.e., MCPES; Soini et al., 2014), other scales have considered it key to ensure acceptable psychometric properties of a scale (i.e., EDMCQ-PE; Milton et al., 2018). However, to our knowledge, no scales have been evaluated for invariance across educational levels.

Based on the above, the main aim of the current study was to assess the psychometric properties of students’ perceived need-supportive and need-thwarting Teaching Interpersonal Style Questionnaire in PE (TISQ-PE) in primary and secondary educational settings. Specifically, we aimed to gather evidence of validity regarding content, factorial, convergent discriminant, and nomological aspects, as well as reliability and factorial invariance across sex (male and female) and educational stage (primary and secondary). Based on this objective, the central hypothesis was that this instrument would provide adequate evidence of its psychometric quality in the target population.

## Materials and methods

### Participants

A total of 654 students with a mean age of 11.96 years (*SD* = 1.95; range = 10–16 years old; 317 boys and 337 girls) from eight primary (5^th^ grade = 174, and 6^th^ grade = 211) and secondary (7^th^ grade = 57, 8^th^ grade = 50, 9^th^ grade = 58, 10^th^ grade = 52, and 11^th^ grade = 52) schools in south-western Spain agreed to participate (see Table 1). The sample was selected using a non-probabilistic, convenience approach, based on schools’ willingness to participate and their geographical location (north–south gradient to ensure representativeness). Inclusion criteria required that students were enrolled in the participating schools, attended PE lessons regularly, and provided parental consent. Exclusion criterion was failure to complete the questionnaires. The schools included both public and semi-private institutions, all of which followed the same national curriculum. Class sizes ranged from 16 to 28 students per class and all PE lessons conducted were based on the current educational law. Participants completed a set of questionnaires in paper-and-pencil format at the end of the school year to make sure that they had a solid perception of the target variables. From an original sample of 662 questionnaires collected, eight (<2%) were excluded because they were incomplete. All remaining questionnaires were fully completed, so no further missing data handling was required.

**Table 1:** Descriptive statistics of TISQ-PE factors by gender and educational stage.

Variables	Gender				Educational stage			
	Female		Male		Primary		Secondary	
	*M*	*SD*	*M*	*SD*	*M*	*SD*	*M*	*SD*
1. Autonomy support	3.37	1.06	3.30	1.08	3.60	1.06	2.98	0.97
2. Competence support	4.02	0.93	3.95	0.94	4.24	0.88	3.62	0.89
3. Relatedness support	3.98	1.02	3.97	0.99	4.27	0.90	3.57	1.01
4. Autonomy thwarting	2.05	1.01	2.09	1.00	1.82	0.95	2.41	0.97
5. Competence thwarting	1.56	0.78	1.57	0.74	1.37	0.60	1.83	0.87
6. Relatedness thwarting	1.48	0.77	1.55	0.87	1.35	0.64	1.75	0.98

### Measures

#### Teachers’ interpersonal style

The Teachers’ Interpersonal Style Questionnaire in Physical Education (TISQ-PE) was elaborated in the present study to provide initial evidence of validity for assessing students’ perception of the teacher’s need-supportive and need-thwarting style. The questionnaire begins with the stem: “In the Physical Education lessons, my teacher…,” and it included 24 items divided into six factors: three to assess students’ perceptions of a supportive teaching style (autonomy, competence, and relatedness support; 12 items) and three to assess students’ perceptions of a thwarting teaching style (autonomy, competence, and relatedness thwarting; 12 items). The students’ perceptions of a supportive teaching style were assessed using the above-mentioned QBPNS-PE (Sánchez-Oliva et al., 2013), which evaluates three correlated factors: autonomy support (four items, e.g., “…often asks about our preferences regarding the activities to be carried out”), competence support (four items; e.g., “…encourages us to trust in our ability to do tasks well”) and relatedness support (four items, e.g., “…encourages good relations between classmates at all times”). To assess the students’ perception of a thwarting teaching style, an adaptation to the PE context of the Coaches’ Interpersonal Style Questionnaire (CIS-Q; Pulido et al., 2018) was used. It assesses three correlated factors: autonomy thwarting (four items, e.g., “…forces me to behave in a certain way”), competence thwarting (four items, e.g., “…proposes situations that make me feel incapable”), and relatedness thwarting (four items, e.g., “…makes me feel not very accepted in this class group”). Students responded to all items on a 5-point scale ranging from 1 (*strongly disagree*) to 5 (*strongly agree*).

#### Needs satisfaction

The Spanish version of the Basic Psychological Needs in Exercise Scale (BPNES; Vlachopoulos and Michailidou, 2006), validated for the PE context by Moreno et al. (2008), was used to assess the students’ perceptions of needs satisfaction. It begins with the stem: “In my Physical Education lesson,” and it has a total of 12 items divided into three correlated factors that represent each one of the basic psychological needs: autonomy satisfaction (four items, e.g., “…We carry out exercises that interesting to me”), competence satisfaction (four items, e.g., “…I carry out the exercises effectively”), and relatedness satisfaction (four items, e.g., “…My relationship with my classmates is friendly”). Students rated all items on a 5-point scale ranging from 1 (*strongly disagree*) to 5 (*strongly agree*). A confirmatory factor analysis (CFA) supported this factor structure,^1^ showing an acceptable model fit: *χ*^2^ = 232.555, *df* = 51; CFI = 0.929; TLI = 0.908; RMSEA = 0.061 (95% CI = 0.025–0.056); SRMR = 0.042. Cronbach’s alpha values also showed acceptable internal reliability for autonomy (*α* = 0.77; *ω* = 0.78), competence (*α* = 0.68; *ω* = 0.69), and relatedness satisfaction (*α* = 0.82; *ω* = 0.83).

#### Needs frustration

The Spanish version of the Psychological Needs Thwarting Scale (PNTS; Bartholomew et al., 2011), developed for the context of PE, was used to assess students’ perceived needs frustration (Leo et al., 2022). It begins with the stem: “In my Physical Education lessons…,” and it includes three correlated factors representing the frustration of each of the basic psychological needs: autonomy frustration (four items, e.g., “…I feel pushed to behave in certain ways”), competence frustration (four items, e.g., “…I have sometimes been told things that made me feel useless”), and relatedness frustration (four items, e.g., “…I feel that other people dislike me”). Students rated all items on a 5-point scale ranging from 1 (*strongly disagree*) to 5 (*strongly agree*). A CFA supported this factor structure, showing good fit indices, *χ*^2^ = 95.101, *df* = 51; CFI = 0.983; TLI = 0.979; RMSEA = 0.030 (95% CI = 0.046–0.065); SRMR = 0.026. Cronbach’s alpha values also showed acceptable internal reliability for autonomy frustration (*α* = 0.73; *ω* = 0.74), competence frustration (*α* = 0.78; *ω* = 0.77), and relatedness frustration (*α* = 0.74; *ω* = 0.74).

### Procedure

First, the research project was approved by the Ethics Committee of the researchers’ University, complying with the standards of the Declaration of Helsinki of 1964 and with the ethical requirements set by the American Psychological Association (APA) (2020). A cross-sectional design was carried out, collecting data in the last third of the academic year 2017/2018 to leave enough time for the students to integrate the subject and the teachers’ teaching style.

To conduct data collection similarly in all the schools, a precise protocol was developed. Firstly, the teachers were informed of the research objectives, and that the results would be used confidentially. Secondly, upon agreeing to participate in the study, a written consent was designed for the participant’s parents or legal guardians, who had to return it signed for the study to begin. It was specified that participation was voluntary, anonymous, and without any type of financial reward. Thirdly, the principal researcher previously explained the objective of the study to the students, underlining that there were no right or wrong answers and that their responses would remain confidential and anonymous. Fourthly, after all the permissions and informed consents had been obtained, data were collected Finally, all participants completed the questionnaire package in the same order, individually, without a time limit during a PE class, and in a comfortable space that allowed them to concentrate on their answers. A research assistant was present during data collection to attend to any questions raised by the participants. The process lasted approximately 10–12 min.

### Data analysis

First, a content analysis was conducted to ensure the validity of the instrument. Items were reviewed and adapted through a qualitative process by experts who independently evaluated them and subsequently discussed them in a think-aloud process. Consensus (≥75% agreement) was required to finalize item wording, ensuring the questionnaire was appropriate for the educational context.

Second, quantitative analyses were conducted using the statistical programs SPSS 19.0 and Mplus 7.0 to process the data. To assess the psychometric properties of the instrument, a CFA was conducted with a covariance matrix using the MLR. The following indices were used to verify the fit of the model: *χ*^2^ (Chi-Square), *df* (degrees of freedom), SRMR (Standardized Root Mean Residual), RMSEA (Root Mean Square Error of Approximation), CFI (Comparative Fit Index), TLI (Tucker-Lewis Index). To evaluate the suitability of the data to the model, scores higher than 0.90 were considered acceptable for the incremental indices such as CFI and TLI (Hu and Bentler, 1999), and values lower than 0.06 for the RMSEA, and 0.08 for the SRMR (Browne and Cudeck, 1993). Factor loadings with values higher than 0.40 were considered appropriate (Hair et al., 2010). Furthermore, evidence of convergent validity was assessed by computing the Average Variance Extracted (AVE) for each factor, with values above 0.50 indicating adequate convergence of the items on their respective constructs (Fornell and Larcker, 1981).

Subsequently, descriptive and reliability (Cronbach’s alpha) analyses of each one of the factors of the questionnaire were conducted. Internal consistency values higher than 0.70 were considered acceptable (Nunnally and Bernstein, 1994). In addition, evidence of discriminant validity was examined using latent correlations between factors; and evidence of nomological validity of the scale was also assessed through the analysis of latent correlations between needs satisfaction and needs frustration, as these variables are closely associated with the target variables (Sánchez-Oliva et al., 2014). Finally, a matrix of variances and covariances was used to test the factorial invariance based on sex (female and male) and educational stage (primary and secondary) through four different models: (1) configural invariance (loading pattern is similar in all groups but the magnitude of all parameters—loadings, intercepts, variances, etc.—may vary); (2) weak invariance (factor loadings/cross-loadings are constrained to be equal to fit the data, and the fit of this model is compared with the baseline model); (3) strong measurement (factor loadings and item intercepts are constrained to be equal to fit the data, and are compared with the weak measurement invariance model); and (4) strict invariance (invariance of the factor loadings/cross-loadings, intercepts, and uniquenesses are constrained to be equal to fit the data and are compared with the strong measurement invariance model).

## Results

### Evidence of content, factorial, and convergent validity

First, to examine the psychometric properties of the TISQ-PE, evidence supporting its content validity was analyzed. To prepare the instrument for the education context, a group of four experts in physical education and educational psychology, aged 33 to 54 years (*M* = 39.25, *SD* = 9.91), performed the adaptation of the items for each factor. The expert group was made up of university professors with extensive experience (10–24 years) in teaching and research (*M* = 14.50, *SD* = 6.45), with more than 130 papers published in high-impact journals and a broad experience in the validation of questionnaires containing variables related to educational contexts. Therefore, the authors’ group demonstrated experience in the creation and adaptation of methodological scales and in the context in which the evidence of validity would be collected. Each researcher independently adapted the sports terms to the educational context. Subsequently, a meeting was carried out through a think-aloud protocol (Jääskeläinen, 2010) among the group of experts. All versions were discussed and merged into one through consensus. Seventy-five percent agreement among the four investigators was required to accept the wording of each item. The final set of items selected for the questionnaire is presented in the Appendix.

**Figure 1:**
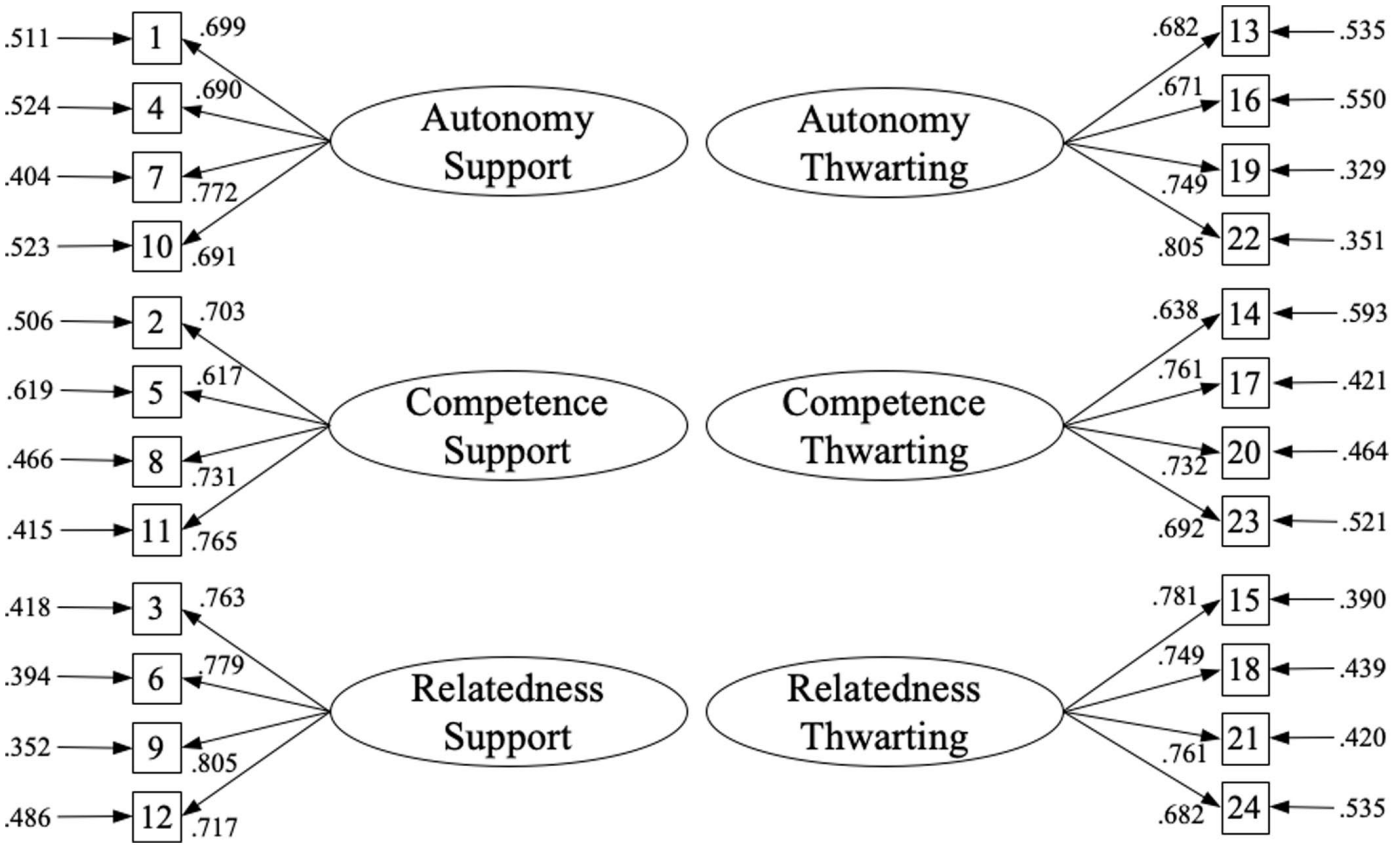
Confirmatory factor analysis of the TISQ-PE.

Second, a CFA was conducted to verify the factorial structure of the questionnaire with a first-order model with the six correlated factors: three to assess students’ perceptions of a supportive teaching style (autonomy, competence, and relatedness support) and three to assess students’ perceptions of a thwarting teaching style (autonomy, competence, and relatedness thwart). Figure 1 shows the factorial loads of each of the items on their factor, with adequate values in all cases (*λ* = 0.617 – 0.805). Furthermore, the values of the fit indices were acceptable in all cases: *χ*^2^ = 453.580, *df* = 237; CFI = 0.957; TLI = 0.950; RMSEA = 0.037 (95% CI = 0.033–0.043); and SRMR = 0.039.

Third, to examine evidence of convergent validity, the AVE values for each factor ranged from 0.54 to 0.61, indicating adequate convergence of items on their respective constructs.

### Descriptive statistics and internal consistency

Table 2 presents the descriptive statistics (i.e., item means and standard deviations) of the teaching supportive and thwarting style factors. The means were below the central values in the thwarting factors and above the central values in the supportive factors. Table 1 shows the internal consistency values of all the factors, where scores higher than 0.70 were observed in all cases.

**Table 2:** Descriptive statistics, internal consistency, discriminant validity, and nomological validity.

Variables	1	2	3	4	5	6	7	8	9	10	11	12
1. Autonomy support	—						0.58^**^	0.43^**^	0.44^**^	−0.18^**^	−0.17^**^	−0.15^**^
2. Competence support	0.65^**^	—					0.45^**^	0.56^**^	0.48^**^	−0.11^**^	−0.26^**^	−0.17^**^
3. Relatedness support	0.64^**^	0.76^**^	—				0.33^**^	0.50^**^	0.52^**^	−0.16^**^	−0.22^**^	−0.23^**^
4. Autonomy thwarting	−0.26^**^	−0.25^**^	−0.29^**^	—			−0.28^**^	−0.24^**^	−0.25^*^	0.36^**^	0.41^**^	0.40^**^
5. Competence thwarting	−0.27^**^	−0.34^**^	−0.35^**^	0.50^**^	—		−0.24^**^	−0.23^**^	−0.25^**^	0.29^**^	0.41^**^	0.39^**^
6. Relatedness thwarting	−0.28^**^	−0.38^**^	−0.36^**^	0.51^**^	0.69^**^	—	−0.16^**^	−0.22^**^	−0.20^**^	0.29^**^	0.34^**^	0.39^**^
*M*	3.33	3.98	3.97	2.07	1.56	1.51	3.11	3,81	3.98	2.02	1.92	1.93
*SD*	1.06	0.94	1.01	1.00	0.76	0.82	0.99	0.83	0.96	0.87	0.94	0.94
α	0.81	0.78	0.85	0.82	0.79	0.84	0.77	0.68	0.82	0.73	0.78	0.74
ω	0.82	0.78	0.84	0.83	0.81	0.85	0.78	0.69	0.83	0.74	0.77	0.74

7. Autonomy satisfaction; 8. Competence satisfaction; 9. Relatedness satisfaction; 10. Autonomy frustration; 11. Competence frustration; 12. Relatedness frustration. *p < 0.05, **p < 0.01.

### Evidence of discriminant and nomological validity

Regarding evidence of discriminant validity, Table 1 shows the correlations between latent factors. Positive correlations were found among students’ perceptions of supportive teaching style factors (*r* = 0.64–0.76), and also among students’ perceived need-thwarting style factors (*r* = 0.50–0.69). In addition, negative relationships were found among students’ perceptions of supportive and thwarting style factors (*r* = −0.25 to −0.38).

Regarding evidence of nomological validity, results showed positive correlations between students’ perceptions of a supportive teaching style and needs satisfaction, and between a thwarting teaching style and needs frustration. In contrast, negative relationships were found between a supportive style and needs frustration, and between a thwarting style and needs satisfaction.

### Evidence of factor invariance

Invariance of the factor structure was assessed based on sex (male and female) and educational stage (primary and secondary stage) using a multigroup analysis (see Table 3). Results showed that the designed instrument performs similarly in each group. This analysis confirmed that the psychometric properties of the instrument do not vary either for sex or educational stage. Thus, the possible differences between the unconstrained model (Model 1) and the nested models (invariance models) can be tested. Regarding the invariance analysis based on sex, confirmatory factorial models were conducted independently, obtaining appropriate fit index values for male and female models. Both the unconstrained model and the three invariant models showed an adequate fit. In accordance with Cheung and Rensvold (2002), the increase of the CFI and the TLI was lower than 0.01. Therefore, the TISQ-PE can be considered invariant based on sex.

**Table 3:** Factor invariance analysis.

Models	*χ*^2^	Δ*χ*^2^	df	*p*	CFI	ΔCFI	TLI	ΔTLI	RMSEA	ΔRMSEA	SRMR	ΔSRMR
Sex												
Model 0. Females	363.243	—	237	<0.001	0.952	—	0.944	—	0.040	—	0.046	—
Model 0. Males	414.963	—	237	<0.001	0.932	—	0.921	—	0.049	—	0.050	—
Model 1. Configure invariance	777.122	—	474	<0.001	0.942	—	0.932	—	0.044	—	0.048	—
Model 2. Weak invariance	795.159	18.037	492	<0.001	0.942	0.000	0.935	0.003	0.043	−0.001	0.051	0.000
Model 3. Strong invariance	817.171	22.012	516	<0.001	0.942	0.000	0.938	0.003	0.042	−0.001	0.051	0.000
Model 4. Strict invariance	817.171	0.000	516	<0.001	0.942	0.000	0.938	0.000	0.042	0.000	0.051	0.000
Education stage												
Model 0. Primary	414.858	—	237	<0.001	0.937	—	0.927	—	0.044	—	0.047	—
Model 0. Secondary	414.898	—	237	<0.001	0.914	—	0.901	—	0.053	—	0.059	—
Model 1. Configure invariance	829.459	—	474	<0.001	0.929	—	0.916	—	0.048	—	0.052	—
Model 2. Weak invariance	853.361	23.902	492	<0.001	0.926	−0.003	0.917	0.001	0.047	0.001	0.057	0.005
Model 3. Strong invariance	915.468	62.107	509	<0.001	0.917	−0.009	0.910	−0.007	0.049	0.002	0.099	0.042
Model 4. Strict invariance	915.468	0.000	509	<0.001	0.916	−0.001	0.909	−0.001	0.054	0.005	0.099	0.000

Standardized factor loadings are shown to the left of the indicators, and measurement errors to the right.

Regarding the invariance analysis based on educational stage, confirmatory factor models were conducted independently, obtaining appropriate fit index values for primary and secondary students (see Table 3). Similarly, the unconstrained model and the three invariant models presented adequate fit indices, and the increase in the CFI and TLI was lower than 0.01 (Cheung and Rensvold, 2002). Therefore, the TISQ-PE can be considered invariant based on the educational stage.

## Discussion

The current study aimed to evaluate the psychometric properties of the TISQ-PE in primary and secondary educational settings, including evidence of content, factorial, convergent, discriminant, and nomological validity, and reliability, as well as its factorial invariance across sex (male and female) and educational stage (primary and secondary). Results provided evidence of validity and reliability for the instrument to assess primary and secondary education students’ perceptions of their PE teachers’ interpersonal teaching style. Results also showed that the instrument was invariant for boys and girls.

Regarding factorial structure, the first-order 6-factor correlated CFA showed acceptable model fit indexes (CFI and TLI >0.900; SRMR and RMSEA <0.060) and significant factorial loadings for all latent factors were also found (*λ* > 0.60; *p* < 0.05). Evidence of convergent validity was also supported, with all AVE values exceeding the recommended threshold of 0.50 (Fornell and Larcker, 1981). These results align with SDT, which differentiates between needs supportive and needs thwarting strategies (Ahmadi et al., 2023), and confirmed a factorial structure that differentiated the “bright” (supportive; Sánchez-Oliva et al., 2013) and “dark” (thwarting; Pulido et al., 2018) side of each of the three basic psychological needs (Vasconcellos et al., 2020). Also, the results of the current study are in line with those found in the sports context (Pulido et al., 2018), where three factors to define supportive strategies and three factors to define thwarting strategies were also found. These findings support the utility of the TISQ-PE for assessing both motivational sides in PE classrooms.

Regarding interfactor correlations, both the supportive and thwarting factors were positively associated with each other (*r* between 0.42 and 0.74), suggesting evidence of discriminant validity for the questionnaire. Negative associations between supportive and thwarting factors further support the scale’s capacity to distinguish between different motivational strategies. This is an important finding, as previous studies also evaluated the factorial structure of the multidimensional measures of interpersonal style (Sánchez-Oliva et al., 2013; Stenling et al., 2015), obtaining a high interfactor correlation. Furthermore, supportive and thwarting factors were negatively associated, in line with previous studies that also included the “bright and dark” sides of interpersonal style (Pulido et al., 2017; Rocchi et al., 2017; Stenling et al., 2015). The present study also found acceptable reliability of the six factors of the TISQ-PE, with Cronbach’s alphas greater than 0.70 (Nunnally and Bernstein, 1994), confirming the instrument’s reliability.

Evidence of nomological validity was also assessed, testing the associations with needs satisfaction and frustration, and the results confirmed it. Students who perceived a supportive teaching style showed higher needs satisfaction and lower needs frustration, whereas students who perceived a thwarting teaching style showed lower needs satisfaction and higher needs frustration. These results are in line with previous studies within the PE context that also found that teachers’ interpersonal style was a significant predictor of basic psychological needs satisfaction or frustration (see Howard et al., 2025; Vasconcellos et al., 2020).

The study also aimed to evaluate the invariance of the scale across sex and educational stage, and the results confirmed both aspects. The tested model without constraints showed adequate fit to the data, and no substantial changes in the fit indices were observed with increasing constraints. Furthermore, the increase of CFI and TLI was lower than 0.01 (Cheung and Rensvold, 2002). Overall, these findings confirmed that the tested model of this instrument was invariant across primary and secondary stages, and across boys and girls. Therefore, it can be used indistinctly with boys and girls, as well as with primary and in secondary education students. Regarding sex, these results are in line with those presented by Sánchez-Oliva et al. (2013), where the QBPNS-PE was found to be invariant across sex. The present research extends Pulido et al.’s (2018) study on the CIS-Q and Sánchez-Oliva et al.’s (2013) study on the QBPNS-PE because they did not evaluate the invariance across sex and educational stage, respectively. Thus, all the analyses conducted indicate that the TISQ-PE shows promising psychometric properties and highlights its applicability for both boys and girls, as well as across primary and secondary educational stages, making it useful for researchers and educators. Further analyses are needed to fully confirm its validity.

To our knowledge, two questionnaires have been previously designed to assess the teacher’s interpersonal style (i.e., QBPNS-PE and MCPES). However, both share the same limitation: they only assess the “bright” side of motivation (i.e., supportive style). Therefore, the TISQ-PE extends the trend of previous instruments to include both the “bright” and the “dark” side of the teachers’ interpersonal style, through the analysis of strategies to support and thwart students’ basic psychological needs. This instrument fills an important gap in the measurement of teachers’ interpersonal style, offering researchers and teachers a comprehensive tool to understand classroom motivational processes and to examine their potential impact on students’ outcomes in PE. Another strength of this instrument is that it combines brevity with psychometric integrity (Appleton et al., 2016) which makes it an agile and reliable instrument for researchers and scholars.

Finally, several limitations must also be acknowledged. Firstly, the questionnaire assesses students’ perceptions of their teacher’s interpersonal style but no objective evaluation of the teacher’s behavior was conducted. Future studies should provide evidence of criterion-related validity by assessing the association between the scale’s scores and objectively measured behaviors. Secondly, a cross-sectional study was conducted at a specific time. It would be interesting to use the TISQ-PE at different times of the school year to assess its temporal stability. Longitudinal designs would provide greater insight into how students’ responses fluctuate over time. Thirdly, the participants in this study were primary and secondary school students in Spain, which limits the possibility of generalizing the results. Future studies are needed to test the factor structure of the instrument in different countries and different languages. In addition, evidence of cross-cultural validation could be performed to test the instrument’s invariance across different cultures.

In conclusion, the present study has demonstrated evidence of validity and reliability for the TISQ-PE as an instrument to assess primary and secondary education students’ perceptions of their teachers’ interpersonal style in the PE class, evaluating the support and thwarting of the students’ basic psychological needs (competence, autonomy, and relatedness). This research provides a new scale that assesses multiple dimensions of teachers’ interpersonal style, considering the bright and dark sides (supportive and thwarting styles) of motivation and providing a practical tool for researchers and teachers. Furthermore, this scale can help gain an in-depth understanding of the specific role that each type of PE teacher’s interpersonal style plays in the development and maintenance of students’ psychological needs, types of motivation, and outcomes in PE.

## Data Availability

The raw data supporting the conclusions of this article will be made available by the authors, without undue reservation.

## Footnotes

1 All CFA models were estimated using the Maximum Likelihood Robust (MLR) estimator, which is robust to non-normality.
