# Supplementary material for: Validation of the teaching interpersonal style questionnaire in physical education
Source: Front Psychol. 2025 Nov 21;16:1702118. doi: 10.3389/fpsyg.2025.1702118 (PMC12679380; doi:10.3389/fpsyg.2025.1702118)
Supplement: Supplementary file 1 [file Supplementary_file_1.docx]

**Appendix**

| Teaching Interpersonal Style Questionnaire in Physical Education (TISQ-PE)  English version | Cuestionario del Estilo Interpersonal del Docente en Educación Física  (CEID-EF).  Spanish version |
| --- | --- |
| **In Physical Education, my teacher…**  1. …prevents me from taking decisions about the way I study.  2. …sets up situations that make me feel incapable.  3. …shows rejection towards me, in certain occasions.  4. …requires me to do things in a certain way.  5. …makes me feel incompetent, sometimes.  6. …is distant, sometimes.  7. …forces me to behave in a certain way.  8. …sets up tasks and situations that make me feel awkward.  9. …creates an atmosphere that I do not like.  10. …forces me to accept a way of teaching that I do not agree with.  11. …does not give me opportunities to show my potential.  12. …makes me feel little accepted within this class group.  13. …often asks us about our preferences regarding the activities to be performed.  14. …encourages us to trust in our abilities to do the tasks well.  15. …encourages good relations between classmates at all times.  16. …tries to give us choice when performing the activities.  17. …proposes activities adjusted to our skill level.  18. …favors a good atmosphere among the classmates.  19. …takes into account our opinions when designing the lessons.  20. …always tries to help us achieve the goals set in the activities.  21. …tries that all students feel integrated.  22. …allows us to make decisions during task performance.  23. …favors learning and content improvement.  24. …helps us solve conflicts friendly. | **En las clases de Educación Física, mi profesor…**   1. …me impide tomar decisiones respecto al modo que estudio. 2. …me propone situaciones que me hacen sentir incapaz. 3. …en determinadas ocasiones tiene comportamientos de rechazo hacia mí. 4. …me exige a hacer las cosas de una determinada manera. 5. …a veces me hace sentir incompetente. 6. …en ocasiones es indiferente conmigo. 7. …me fuerza a seguir una determinada forma de estar en su clase. 8. …me propone tareas y situaciones que me hacen sentir torpe. 9. …crea un ambiente en el grupo de clase que no me agrada. 10. …me obliga a aceptar una forma de dar clase que yo no comparto. 11. …no me da oportunidades para demostrar mi potencial. 12. …hace que me sienta poco aceptado en este grupo de clase. 13. …nos pregunta a menudo sobre nuestras preferencias con respecto a las actividades a realizar. 14. …nos anima a que confiemos en nuestra capacidad para hacer bien las tareas. 15. …favorece en todo momento las buenas relaciones entre los compañeros/as de clase. 16. …trata de que tengamos libertad a la hora de realizar las actividades. 17. …nos propone actividades ajustadas a nuestro nivel. 18. …favorece el buen ambiente entre los compañeros/as de clase. 19. …tiene en cuenta nuestra opinión en el desarrollo de las clases. 20. …siempre intenta que consigamos los objetivos que se plantean en las actividades. 21. …busca que todos los alumnos/as nos sintamos integrados. 22. …nos deja tomar decisiones durante el desarrollo de las tareas. 23. …favorece el aprendizaje y la mejora de los contenidos de la asignatura. 24. …nos ayuda a resolver los conflictos amistosamente. |

*Note.* Autonomy Thwarting: 1, 4, 7, 10; Competence Thwarting: 2, 5, 8, 11; Relatedness Thwarting: 3, 6, 9, 12; Autonomy Support: 13, 16, 19, 22; Competence Support: 14, 17, 20, 23; Relatedness Support: 15, 18, 21, 24.
